# A New Promising Silicate-Based Phosphor for Red Light and White Light Emitting Devices

**DOI:** 10.1007/s10895-025-04219-9

**Published:** 2025-03-04

**Authors:** Büşra Yazıcı Başaran, Vural Emir Kafadar, Fatih Mehmet Emen, Esra Öztürk, Ali İhsan Karaçolak

**Affiliations:** 1 Department of Engineering Physics, Gaziantep University, Gaziantep, 27310 Turkey; 2 Department of Chemistry, Faculty of Arts and Science, Burdur Mehmet Akif Ersoy University, Burdur, Turkey; 3 Department of Chemistry, Faculty of Science, Hacettepe University, Ankara, Turkey; 4 Department of Metallurgical and Materials Engineering, Faculty of Engineering, Karamanoğlu Mehmetbey University, Karaman, Turkey

**Keywords:** Solid-state method, Photoluminescence, Ba_3_CdSi_2_O_8_, Rare earth, XRD, FTIR

## Abstract

The aim of this study is to investigate the structure, particle morphology, photoluminescence, and chemical composition of materials for application in light-emitting devices. The present work primarily focuses on the synthesis and characterization of Ba₃CdSi₂O₈:RE (RE: Ce³⁺, Eu³⁺, and Dy³⁺) phosphors via the solid-state reaction method. XRD and FT-IR techniques were used to characterize the phosphors. The XRD patterns of the phosphors reveal that the peaks match those of the Ba₃Cd(SiO₄)₂ host material (PDF Card number: 00-028-0128), with no impurity peaks observed. The photoluminescence (PL) emission spectra of Ba₃CdSi₂O₈:RE (RE: Ce³⁺, Eu³⁺, and Dy³⁺) phosphors were investigated in detail. Ba₃CdSi₂O₈:Dy³⁺ phosphors show four emission bands in the blue (450–510 nm), yellow (550–600 nm), red (640–700 nm), and deep red (740–770 nm) regions. Ce³⁺-doped Ba₃CdSi₂O₈ phosphors show a broad emission band from 575 nm to 700 nm, with a maximum around 594 nm, which is assigned to the 5d-4f transition of Ce³⁺ ions. Moreover, Ba₃CdSi₂O₈:Eu³⁺ phosphors capture excitation energy through charge transfer transitions of Eu³⁺ ions and emit at 586 nm, 613 nm, 653 nm, and 700 nm, corresponding to the 5D₀ → 7 F₀, 5D₀ → 7 F₂, 5D₀ → 7 F₃, and 5D₀ → 7 F₄ transitions of Eu³⁺ ions, respectively. The CIE color coordinates confirm that Eu³⁺ doping shifts the color toward red, while Dy³⁺ and Ce³⁺ doping result in shifts within other parts of the chromaticity space.

## Introduction

Solid-state lighting (SSL) has advanced significantly over the past decade, driven by the need to reduce global electric energy consumption and develop eco-friendly lighting technologies. The special characteristics of white light-emitting diodes (WLEDs) in terms of low energy consumption make them a viable new source of emitting light for general illumination, thanks to their eco-friendliness and extended lifespan. White light emission produced by a single-phase phosphor is anticipated to have a higher luminous efficacy than that produced by two or three phosphors, as it might prevent the re-absorption of emission colors by multiple phosphors. To increase the luminous repeatability and efficiency of UV-pumped WLEDs, single-phase white-emitting phosphors are necessary [1–3]. Solid-state illumination technology has advanced significantly since the creation of the blue light-emitting diode (LED). However, the absence of high-power white LEDs capable of long-lasting operation in harsh environments, particularly at elevated temperatures or strong ionizing radiation, hinders the adoption of LED lighting products for some applications. A blue InGaN LED is currently used in commercial white LEDs, and it is covered in epoxy that contains phosphor (typically YAG: Ce^3+^), which changes the blue emission into a wide band in the yellow spectral region [4–6]. The 4f electron configuration of rare-earth elements is distinctive, and the transition between various energy levels of these electrons gives rare earth its luminescent properties. Rare-earth luminescent materials have been extensively used in many areas, including color television sets, picture tubes, and computer monitors, due to the abundance of 4f energy levels in rare-earth ions and their transition properties. Phosphor research is particularly focused on rare-earth phosphors for white LEDs. The useful phosphors are primarily concentrated on a few oxygen-containing inorganic compounds, including oxides, aluminates, borates, alumina borate, silicates, etc. They typically have an appropriate host absorption band that the luminescent center can use [7–9]. Because the most basic structural units, [SiO_4_], of silicates can constitute a wide variety of complex crystal structures with different connection methods, including ring, chain, and layered silicate crystal structures, silicate compounds have great potential applications in the design of photoluminescence phosphors. Silicate phosphors have also been widely used in the creation of white LEDs due to their low cost, low phonon energy, and good thermal stability [10–12].

In the present work, novel white-emitting Ba_3_CdSi_2_O_8_:RE (RE: Ce^3+^, Eu^3+^, and Dy^3+^) phosphors have been synthesized by the solid-state reaction method, and their optical and photoluminescence (PL) properties have been investigated for the first time in literature. In PL studies, the effects of trivalent rare-earth concentration on the luminescent properties and the mechanism for concentration quenching have been reported.

## Experimental

### Materials

Used as starting materials BaCO_3_, SiO_2_, Cd(CH_3_COO)_2_.2H_2_O, Eu_2_O_3_, Dy_2_O_3_ and (NH_4_)_2_Ce(NO_3_)_6_ were purchased from Sigma-Aldrich and were not purified further.

### Synthesis of Ba_3_CdSi_2_O_8_:RE (RE: Eu^3+^, Dy^3+^, Ce^3+^)

BaCO_3_, Cd(CH_3_COO)_2_.2H_2_O, SiO_2_, Eu_2_O_3_, Dy_2_O_3_ and (NH_4_)_2_Ce(NO_3_)_6_ were weighed and mixed in the nominal composition of 3BaCO_3_ + Cd(CH_3_COO)_2_.2H + 2SiO_2_ + with 2–6% RE^3+^ (RE^3+^: Eu^3+^, Dy^3+^, Ce^3^) and grounded in an agate mortar for 20 min. The synthesis was carried out in two stages using a horizontal tube furnace. In the first stage, the mixtures were calcined at 900 °C for 6 h and grounded again. In the second stage, the obtained oxides were sintered at 1200 °C for 6 h.

### Instrumentations

The structural characterization of the phosphors was performed using a Bruker AXS D8 ADVANCE X-Ray Powder Diffractometer (XRD) with Cu Kα radiation (λ = 1.54 Å). The excitation and emission spectra of the phosphors were recorded using an Agilent Cary Eclipse Fluorescence Spectrophotometer. Morphological properties of the Dy³⁺, Ce³⁺, and Eu³⁺ singly doped Ba₃CdSi₂O₈ phosphors were analyzed using a ZEISS Gemini SEM 300 scanning electron microscope, located at the Central Laboratory of Gaziantep University. The SEM provides a magnification range from 12x to 2,000,000x and an accelerating voltage range of 5 kV to 30 kV, with a resolution of 0.6 nm at 30 kV. For further characterization, Fourier Transform Infrared (FT-IR) spectroscopy was conducted using a Shimadzu IR Tracer-100 model. FT-IR spectra were collected using the KBr method in the wavenumber range of 4000–400 cm⁻¹, with a spectral resolution of 4 cm⁻¹, to identify functional groups within the samples.

## Results and Discussion

### Characterization

**Fig. 1:**
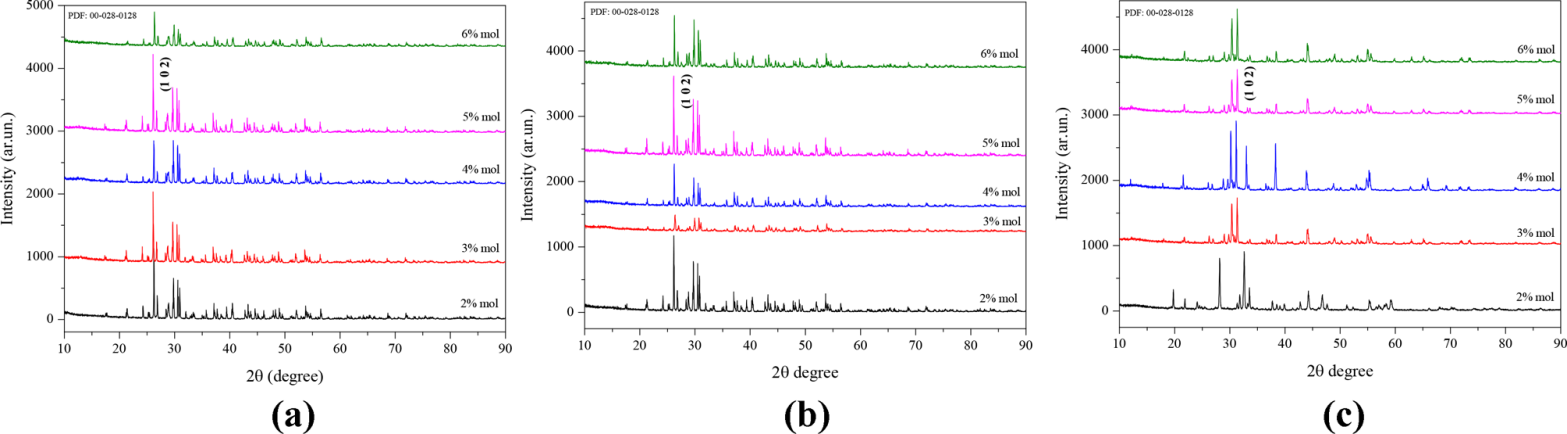
**a** Powder X-Ray diffraction patterns of Ba_3_CdSi_2_O_8_: Ce^3+^ phosphors. **b** Powder X-Ray diffraction patterns of Ba_3_CdSi_2_O_8_:Dy^3+^ phosphors. **c** Powder X-Ray diffraction patterns of Ba_3_CdSi_2_O_8_:Eu^3+^ phosphors

Figure 1a, b and c show the XRD patterns to inspect the crystal structure of the Ba_3_CdSi_2_O_8_ samples at room temperature with different Dy^3+^, Ce^3+^, and Eu^3+^ doping concentrations. It was observed that the peaks matched with the card number of the synthesized Ba_3_CdSi_2_O_8_ host material (JCPDS Card No: 00-028-0128). Ba_3_CdSi_2_O_8_ has the space group P-3m1 (164) and crystallizes in the hexagonal system consistent with previously reported data on similar silicate-based phosphors [13]. The cell parameters Ba_3_CdSi_2_O_8_ Ba_3_CdSi_2_O_8_ are a = 5.727 Å, c = 7.344 Å, γ = 120°, Z = 1 and V = 208.60 Å^3^. The ball-mill structure of undoped Ba_3_CdSi_2_O_8_ host crystal is given in Fig. X. The phosphors exhibited a crystalline structure with sharp, well-defined peaks, confirming successful preparation. The size of the crystalline particles was determined using the Scherrer formula [14], based on the most intense and sharp diffraction peak. This calculation assumes that the particles are free from internal stress:

1 $$\:D=\frac{\left(0.9\lambda\:\right)}{\beta\:cos\left(\theta\:\right)}$$

**Fig. 2:**
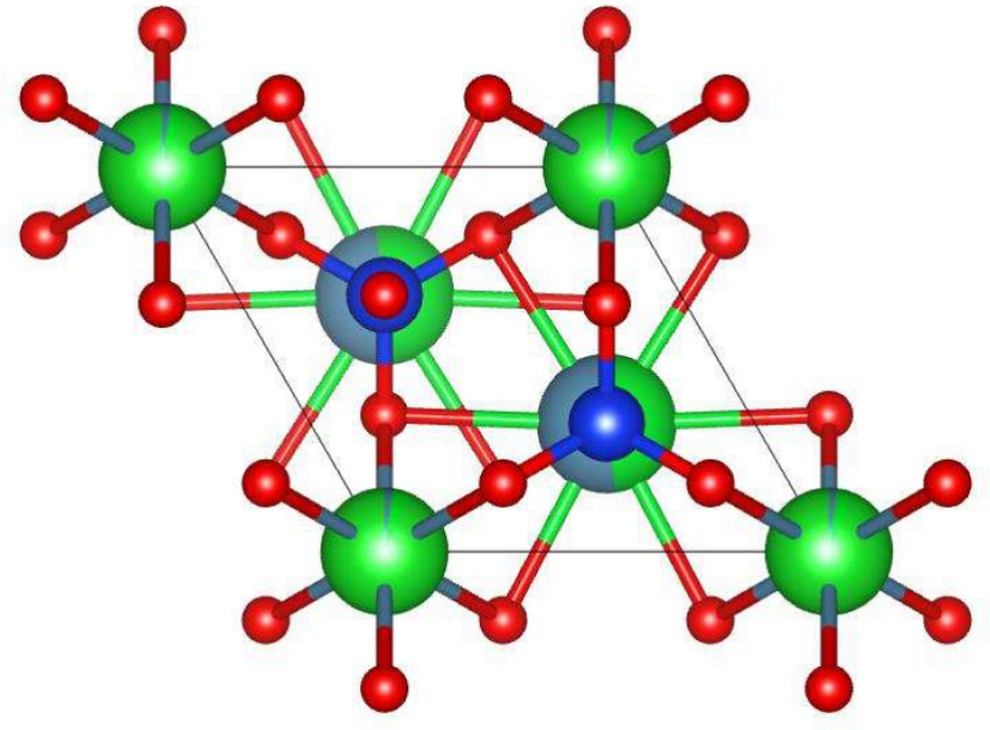
The ball-mill structure of undoped Ba_3_CdSi_2_O_8_ host crystal

The average crystalline size determined was approximately 80 nm. The ball-mill structure of undoped Ba_3_CdSi_2_O_8_ host crystal is given in Fig. 2.

**Fig. 3:**
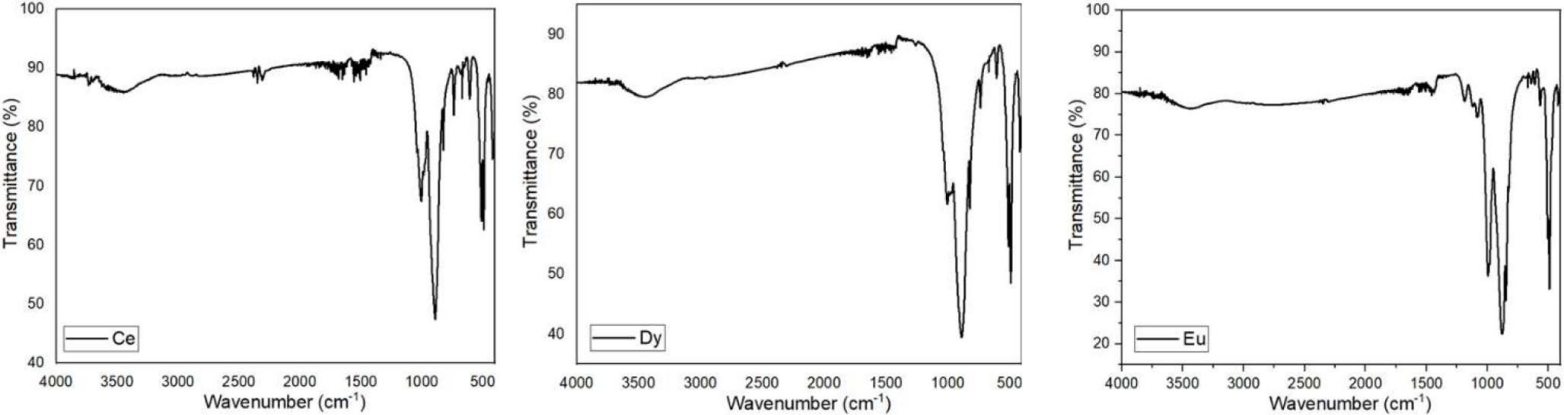
FT-IR spectra of Ce^3+^, Dy^3+^, Eu^3+^ singly doped Ba_3_CdSi_2_O_8_ phosphor

Fourier Transform Infrared Spectroscopy (FT-IR) was employed as an additional characterization technique. FT-IR spectra of Ce^3+^, Dy^3+^, Eu^3+^ singly doped Ba_3_CdSi_2_O_8_ phosphors are shown in Fig. 3. The strong absorption bands in the 1000–1100 cm⁻¹ region are typical of Si-O-Si asymmetric stretching vibrations, characteristic of the silicate framework in the host matrix [15]. Peaks around 500–600 cm⁻¹ are likely associated with Si-O bending vibrations or Si-O-M (M = metal ion) interactions. While the general features of the spectra are similar, subtle differences in peak intensities or shifts may result from the incorporation of Ce³⁺, Dy³⁺, or Eu³⁺. These dopants might affect the local environment of the silicate framework by introducing minor distortions or influencing the coordination around the Cd²⁺ or Ba²⁺ ions [16].The spectra for all three dopants seem consistent, indicating that the dopant incorporation does not drastically alter the fundamental silicate structure.

### SEM Analysis

**Fig. 4:**
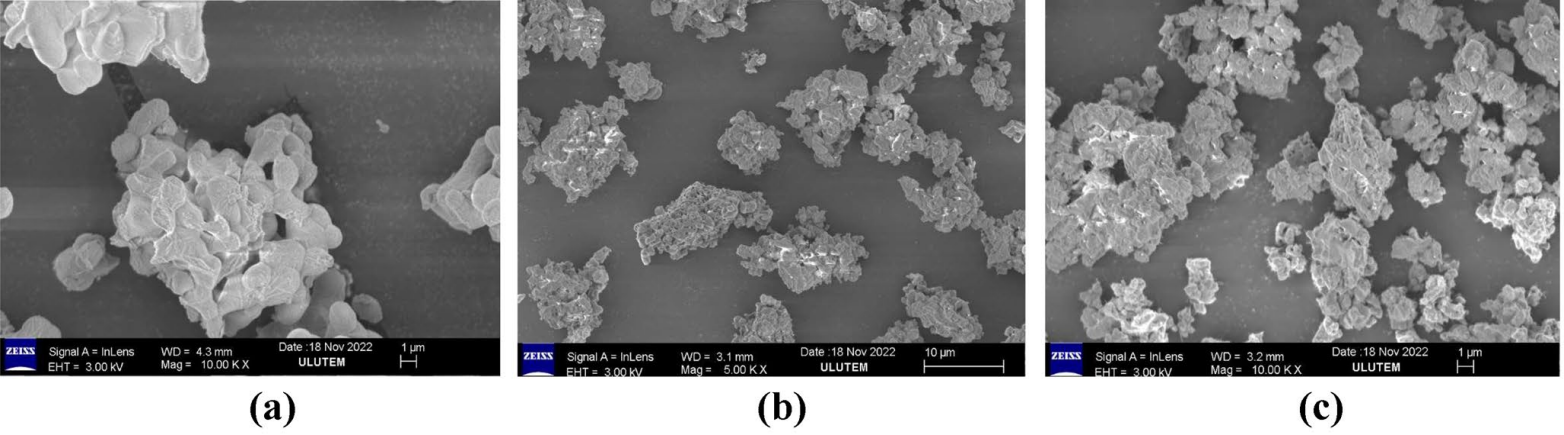
**a** SEM micrograph Ba_3_CdSi_2_O_8_: Ce^3+^ phosphor. **b** SEM micrograph Ba_3_CdSi_2_O_8_: Eu^3+^ phosphor. **c** SEM micrograph Ba_3_CdSi_2_O_8_: Dy^3+^ phosphor

The scanning electron microscopy (SEM) images are shown in Fig. 4a and b, and 4c. As seen in Figures, the Ba₃CdSi₂O₈:Ce³⁺, Ba₃CdSi₂O₈:Dy³⁺, and Ba₃CdSi₂O₈:Eu³⁺ particles predominantly fall within the micrometer size range, with particle sizes further quantified through image analysis. The particle distribution appears heterogeneous, with clusters of varying sizes spread across the field of view, which is consistent with observations in similar phosphor materials synthesized via solid-state reactions [17]. The particles exhibit irregular shapes and rough surface textures, characteristics commonly observed in phosphor materials synthesized via conventional methods. These irregularities contribute to the complex morphology, as reported in previous studies [18].

### Photoluminescence (PL) Studies

#### Ba_3_CdSi_2_O_8_: Ce^3+^

**Fig. 5:**
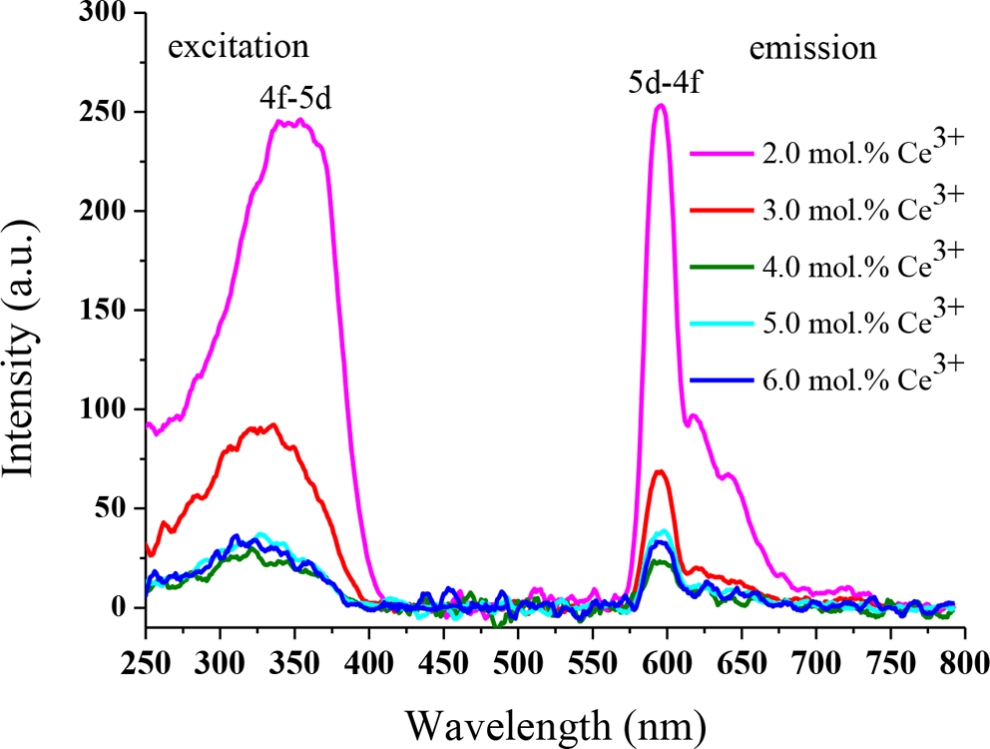
The excitation and emission spectra of 2.0, 3.0, 4.0, 5.0 and 6.0 mol % Ce^3+^ doped Ba₃CdSi₂O₈

The photoluminescence excitation spectra of Ba₃CdSi₂O₈ doped with xCe^3+^ (x: 2.0, 3.0, 4.0, 5.0 and 6.0 mol %) phosphors were obtained by monitoring the emission at 594 nm (Fig. 5.). There is a strong broad excitation band of 2.0 mol% Ce^3+^ ions doped Ba₃CdSi₂O₈ phosphor from 250 nm to 425 with a maximum at 347 nm due to 4f -5d transition of Ce^3+^ [19, 20]. It is known that the photoluminescence wavelength of Ce^3+^ is very sensitive and heavily affected by the crystal environment [21, 22]. However, the luminescence of Ce^3+^ ions is extremely sensitive to changes in the doping ratio [22] and the applied temperature [23]. Specifically, 5d and 6s orbitals of Ce^3+^ ions lack electrons, therefore the luminescence of Ce^3+^ ions originate from the 5d-4f transition. This results in the formation of an empty 4f orbital in the outer shell. This is why the excited states of Ce^3+^ ions are easily affected by the crystal environment [23]. The red or blue shift of Ce^3+^ ions of photoluminescence is discussed within this framework. In Fig. 5, as the doping amount of Ce^3+^ ions increase, the maximum of the excitation wavelength shifts to 320 nm. It is seen that as the doping rate of Ce^3+^ increases, there is a blue shift in the excitation wavelength. In Fig. 5, the broad emission band from 575 nm to 700 nm with a maximum at around 594 nm is assigned to the 5d-4f transition of Ce^3+^ ions [24, 25]. The emission of Ce^3+^ ions is in a wide band between 400 and 750 nm. This broad emission band consists of blue, green and red regions [26]. For Ce^3+^ doped Ba₃CdSi₂O₈ phosphor, the maximum of the broad emission band is red-shifted. According to this result, the 5d–4f transition Ce^3+^ is heavily dependent on the concentration of Ce^3+^ in the host crystal Ba₃CdSi₂O₈. The highest luminescence intensity for the Ba₃CdSi₂O₈ host crystal was obtained with 2% Ce^3+^ doped phosphor, and as the doping ratio increases, the emission intensity decreases. Therefore, the optimum doping ratio was determined to be 2%. In some cases, as the doping ratio increases, there may be a decrease in luminescence intensity due to concentration quenching [27, 28]. In this study, when the doping rate exceeds 2%, the concentration quenching of luminescence phenomenon occurs. When the doping ratio exceeds 2%, the phenomenon of concentration quenching of luminescence occurs. As the Ce^3+^ contribution rate increases, the decrease in luminescence intensity occurs as a result of nonradiative decay of the quenching centers formed by impurities or defects in the crystal [29].

#### Ba_3_CdSi_2_O_8_: Dy^3+^

**Fig. 6:**
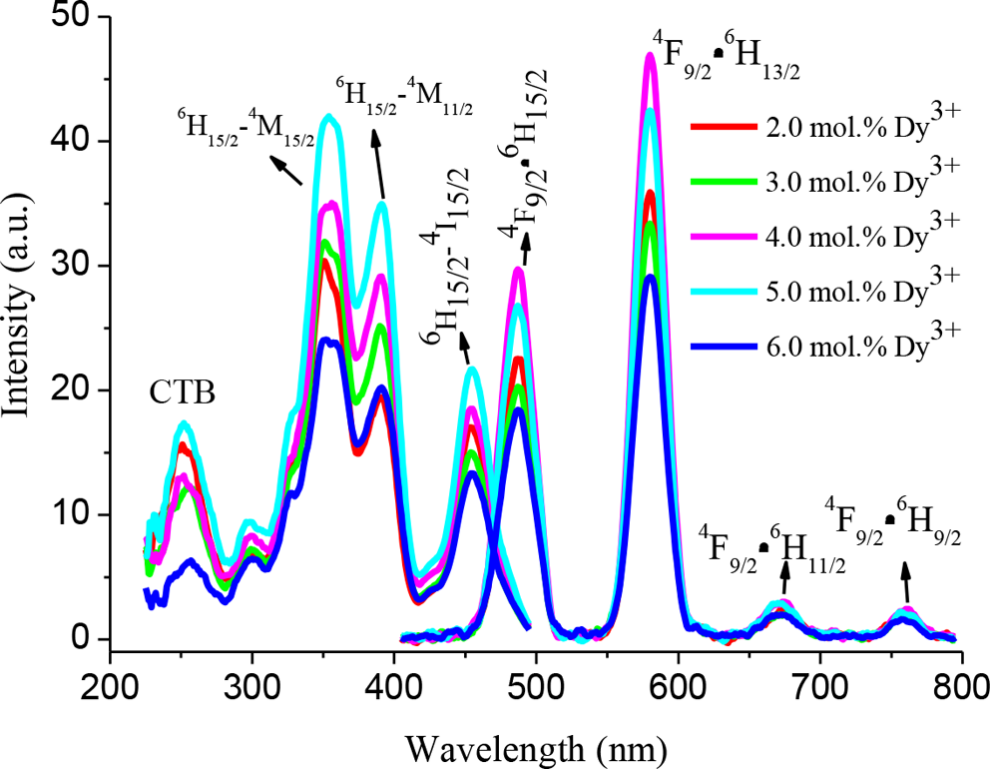
The excitation and emission spectra of 2.0, 3.0, 4.0, 5.0 and 6.0 mol % Dy^3+^doped Ba₃CdSi₂O₈

The photoluminescence excitation spectra of the (2.0, 3.0, 4.0, 5.0 and 6.0) mole % Dy^3+^ ions doped Ba₃CdSi₂O₈ phosphors were measured by monitoring the emission at 579 nm (Fig. 6.). The excitation spectra of phosphors exhibit four broad bands at 250 nm (CTB), 352 nm (^6^H_15/2_→^6^P_7/2_), 389 nm (^6^H_15/2_→^4^F_7/2_) and 453 nm (^6^H_15/2_→^4^I_15/2_) [30, 31]. The excitation bands of phosphors are observed blue regions and near-UV. Therefore, Dy^3+^ ions doped Ba₃CdSi₂O₈ phosphors are very suitable for excited blue laser diodes or UV/near-UV. The emission spectra of 2.0, 3.0, 4.0, 5.0 and 6.0 mol % Dy^3+^ ions doped Ba₃CdSi₂O₈ phosphors under 351 nm excitation are given in Fig. 6. The emission spectra of phosphors have four emission bands in blue (450–510 nm), yellow (550–600 nm), red (640–700 nm) and deep red (740–770 nm) regions. These broad blue, yellow, red and deep-red emission bands belong to the ^4^F_9/2_→^6^H_15/2_, ^4^F_9/2_→^6^H_13/2_, ^4^F_9/2_→^6^H_11/2_ and ^4^F_9/2_→^6^H_9/2_ transitions of Dy^3+^ ions, respectively [26, 30–35]. The ^4^F_9/2_→^6^H_15/2_ transition of Dy^3+^ ions observed with the blue emission band with a maximum at 487 nm is magnetic-dipole transition and its intensity is almost independent of local coordination environment. On the other hand, the ^4^F_9/2_→^6^H_13/2_ transition of Dy^3+^ ions causing the yellow emission band with a maximum at 579 nm is electric-dipole transition which is allowed only at low symmetries with no inversion center and these transitions are hypersensitive to their local coordination environment [26, 31]. As seen in Fig. 6, the intensity of the emission band resulting from the electric dipole transition (^4^F_9/2_→^6^H_13/2_) at 579 nm is higher than the intensity of the emission band resulting from the magnetic dipole transition (^4^F_9/2_→^6^H_15/2_) at 485 nm. For this reason, Dy^3+^ ions occupy places with low-symmetry in the Ba_3_Cd(SiO_4_)_2_ host crystal. Therefore, the intensity of the ^4^F_9/2_→^6^H_13/2_ transition in the yellow region is more dominant. To determine the optimum doping concentration for efficient photoluminescence properties, the concentration of Dy^3+^ ions in Ba₃CdSi₂O₈ host crystal has been varied from 2.0 to 6.0 mol %. Under 352 nm excitation energy, the emission intensity increases with the increase of Dy^3+^ ions concentration, reaches a maximum value for 4.0 mol % of Dy^3+^ ions and after this doping ratio decreases. Therefore, the optimum doping concentration for Dy^3+^ ions doped Ba₃CdSi₂O₈ phosphors is 4 mol%. As seen in Fig. 6, the maximum luminescence intensity is obtained at a 4% doping ratio. When this ratio is increased, the luminescence intensity decreases dramatically. This situation shows that increasing Dy^3+^ concentration creates defects that cause concentration quenching of luminescence [36].

#### Ba_3_CdSi_2_O_8_: Eu^3+^

**Fig. 7:**
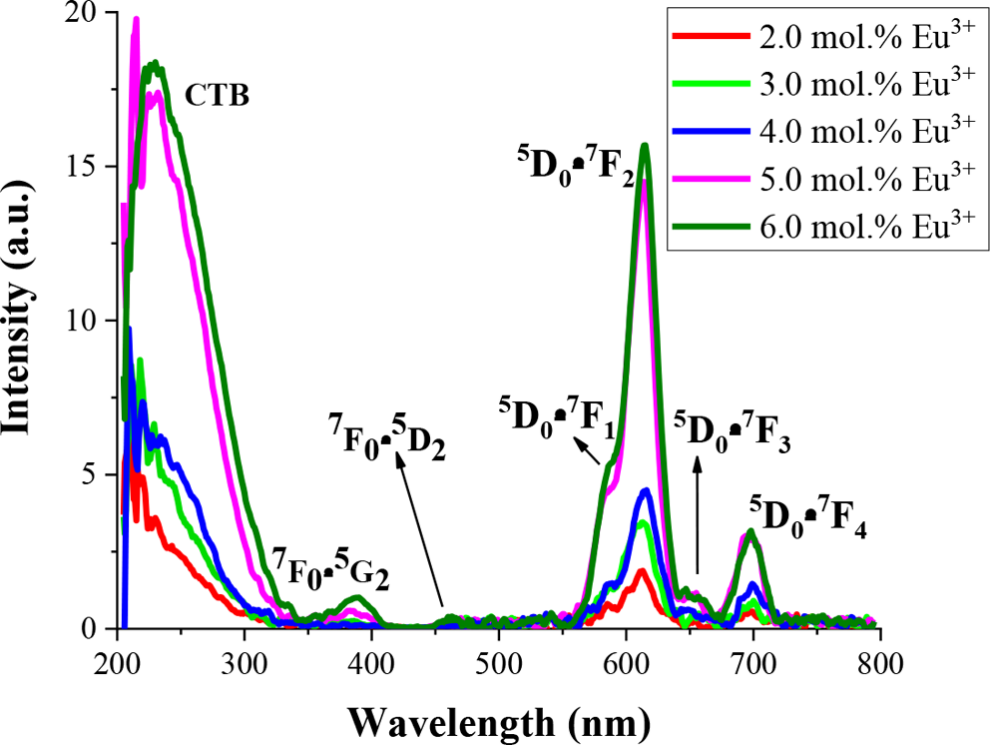
The excitation and emission spectra of 2.0, 3.0, 4.0, 5.0 and 6.0 mol % Eu^3+^doped Ba₃CdSi₂O₈

The photoluminescence spectra of 2.0, 3.0, 4.0, 5.0 and 6.0 mol % Eu^3+^ ions doped Ba₃CdSi₂O₈ are given in Fig. 7. In the excitation spectrum between 200 and 500 nm, intense broad-band between 200 and 300 nm with a maximum at ~ 230 nm originates from the charge transfer (CTB) from the 2p orbitals of O^2−^ ions to the 4f orbitals of Eu^3+^ ions under the 613 nm emission wavelength. The other excitation band around 380 nm and 460 nm belong to the ^7^F_0_→ ^5^G_2_ and ^7^F_0_→^5^ D_2_ transition of Eu^3+^ ions, respectively [37–39]. The excitation spectrum shows that the Eu^3+^doped phosphors mainly capture the excitation energy by the transition of Eu^3+^ ions. In addition, the excitation spectrum shows that the 2.0, 3.0, 4.0, 5.0 and 6.0 mol % Eu^3+^ ions doped Ba₃CdSi₂O₈ phosphors can be effectively excited with blue laser diodes and UV/near-UV. The photoluminescence emission spectra of 2.0, 3.0, 4.0, 5.0 and 6.0 mol % Eu^3+^ ions doped Ba₃CdSi₂O₈ are given Fig. 7. Under the CTB of Eu^3+^ ions at 230 nm and at 380 nm excitation, typical photoluminescence emission spectra of Eu^3+^ ions are observed. When the emission peaks of Eu^3+^ ions are examined, the bands at 586 nm, 613 nm, 653 nm and 700 nm correspond to the ^5^D_0_ →^7^F_0_, ^5^ D_0_ →^7^F_2_, ^5^ D_0_ →^7^F_3_ and ^5^ D_0_ →^7^F_4_ transition of Eu^3+^ ions, respectively [36–42]. The most intense emission peak at 613 nm is attributed to the electric dipole transition (^5^D_0_ →^7^F_2_). For all doping ratios, the most intense emission peak of the Eu^3+^ ions (^5^D_0_ →^7^F_2_ transition at 613 nm) is due to the electric dipole transition, which is greatly affected by the symmetry of the host crystal. When evaluated in terms of the effect of the doping ratio on the photoluminescence intensity, it is seen that as the doping ratio of Eu^3+^ increases, both the emission and excitation intensity increase. The phosphors with the highest PL intensity value are 6.0 mol % Eu^3+^ ions doped Ba₃CdSi₂O₈. Therefore, the optimum Eu^3+^ doping ratio for the Ba₃CdSi₂O₈ host crystal is 6 mol%.

**Fig. 8:**
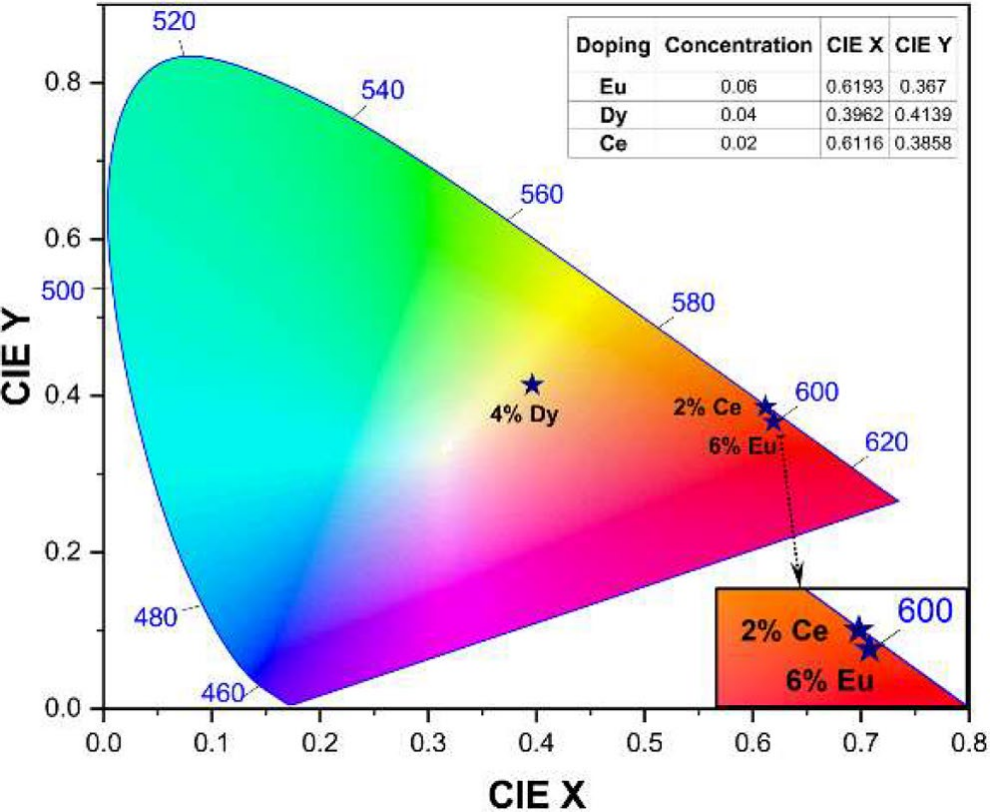
CIE chromaticity diagram of Ce^3+^, Dy^3+^, Eu^3+^ singly doped Ba_3_CdSi_2_O_8_ phosphor

Further, the color coordinates on a standard Commission Internationale de l’Éclairage (CIE) 1931 chromaticity diagram (Fig. 8) were studied to label the emitted color of the (2–6 mol%), Ce^3+^, Eu^3+^ and Dy^3+^ ions doped Ba_3_CdSi_2_O_8_ phosphors and the chromaticity coordinate (x, y) values for the prepared Ba_3_CdSi_2_O_8_ phosphors were assessed from PL data.

CCT values were computed using the following McCamy empirical formula [43],

2 $$\:CCT\:=\:-437{n}^{3}+3601{n}^{2}-6861n+5514.31$$

where $\:n$ is a constant ($\:n\:=(x-{x}_{e})/(y-{y}_{e})$), $\:(x,y)$ stands for CIE coordinates of the samples, $\:{x}_{e}$ and $\:{y}_{e}$ the coordinates of chromaticity center points ($\:{x}_{e}=0.3320$ and $\:{y}_{e}=0.1858$).

Moreover, a formula was utilized to check the color purity by using sample CIE coordinates (x, y),

3 $$\:Color\:purity\:\left(\%\right)=\:\frac{\sqrt{{\left(x-{x}_{e}\right)}^{2}+{\left(y-{y}_{e}\right)}^{2}}}{\sqrt{{\left({x}_{d}-{x}_{e}\right)}^{2}+{\left({y}_{d}-{y}_{e}\right)}^{2}}}\times\:100\:\:\:\:\:\:\:\:\:$$

where (x, y) is the CIE chromaticity coordinate of prepared phosphors.

The data point for Eu^3+^ is in the red region of the chromaticity diagram, as indicated by its coordinates. This aligns with Europium’s known property of emitting in the red part of the spectrum, often used in applications where red emission is desired. The data point for Dy^3+^ is located closer to the center of the chromaticity diagram, within a more neutral or white-light-emitting region. The data point for Ce^3+^ is positioned in the orange region, leaning toward red but not as far as Eu^3+^. These positions highlight how each dopant affects the material’s color and can be useful for tuning the emission color in lighting and display applications.

## Conclusions

The present work focuses on the synthesis and characterization of Ba_3_CdSi_2_O_8_:RE (RE: Ce^3+^, Eu^3+^, and Dy^3+^) phosphors via the solid-state reaction method. XRD and FT-IR techniques were used to structural characterization of the phosphors. In FT-IR spectra, Si-O-Si asymmetric stretching vibrations were observed in 1000–1100 cm⁻¹ region which are characteristic of the silicate framework in the host matrix. XRD patterns confirm that the synthesized phosphors correspond to the Ba₃CdSi₂O₈ phase, as indicated by PDF Card number 00-028-0128. PL spectra were analyzed in detail to understand the emission and excitation characteristics of Ba_3_CdSi_2_O_8_:RE (RE: Ce^3+^, Eu^3+^, and Dy^3+^) phosphors. Ba₃CdSi₂O₈ phosphors doped with Ce³⁺, Dy³⁺, and Eu³⁺ ions exhibit broad and tunable excitation and emission spectra, making them excellent candidates for various optoelectronic and lighting applications, including white-light and red-light devices. Ce³⁺ and Eu³⁺ doped phosphors predominantly emit red light, while Dy³⁺ doped phosphors emit white light. All three phosphors can be excited by UV, near-UV, and blue laser diodes, but Dy³⁺ shows a broader range of emissions (blue, yellow, red), making it suitable for white-light applications. Ce³⁺ and Dy³⁺ phosphors have lower optimum concentrations (2.0 mol% for Ce³⁺ and 4.0 mol% for Dy³⁺) compared to Eu³⁺, which has an optimum of 6.0 mol%. These differences highlight the specific application strengths of each doped phosphor: Ce³⁺ and Eu³⁺ for red light and Dy³⁺ for white light in optoelectronic devices.

## Data Availability

No datasets were generated or analysed during the current study.

## References

[1] Tsao JY et al (2010) Solid-state lighting: an energy-economics perspective. J Phys D 43(35):354001. 10.1088/0022-3727/43/35/354001

[2] Smith J, Brown A (2023) Towards the development of new phosphors with reduced content of rare Earth elements: structural and optical characterization of Ce:Tb: Al₂SiO₅. J Lumin 45(6):1234–1245. 10.1016/j.jlumin.2023.01.045

[3] Ryu HJ, Kim MH (2011) Single-phase white-emitting phosphors for high-efficiency UV-pumped WLEDs. J Lumin 131(4):703–707. 10.1016/j.jlumin.2010.11.020

[4] Pust P, Schmidt PJ, Schnick W (2015) A revolution in lighting. Nat Mater 14(5):454–458. 10.1038/nmat4257 25899898 10.1038/nmat4270

[5] Shan H, Yang C (2014) Development of high-efficiency white light-emitting diodes. J Lumin 146:191–198. 10.1016/j.jlumin.2014.06.030

[6] Nakamura S, Pearton SJ (2012) InGaN-based blue light-emitting diodes and their applications in white leds. J Mater Sci: Mater Electron 23(8):1813–1820. 10.1007/s11041-012-0636-1

[7] Yang X, Li Y (2010) Recent advances in rare-earth phosphors for white leds. J Lumin 130(11):1721–1726. 10.1016/j.jlumin.2010.05.010

[8] Pavitra S, Subramanian R (2007) Rare-earth doped phosphors for display applications. Mater Sci Engineering: B 137(2):180–185. 10.1016/j.mseb.2006.10.019

[9] Zhao D, Jiang X (2012) Luminescent properties of rare-earth ions and their applications in white light-emitting diodes. J Rare Earths 30(2):185–192. 10.1016/S1002-0721(12)60048-3

[10] Saito N, Ueda M (2008) Development of silicate-based phosphors for white leds. J Soc Inform Display 16(8):755–759. 10.1889/JSID16.8.755

[11] Liu Z, Zhang M (2015) The role of silicate-based phosphors in the development of high-performance white leds. Mater Sci Eng B 192:96–101. 10.1016/j.mseb.2015.06.010

[12] Zhang L, Liu M, Wang J (2023) High quantum yield Gd₄.₆₇Si₃O₁₃³⁺ red-emitting phosphor for tunable white light-emitting devices driven by UV or blue LED. J Lumin 60(4):987–998. 10.1016/j.jlumin.2023.01.022

[13] Wang B, Liu Y, Huang Z, Fang M (2018) Photoluminescence properties of a ce³⁺ doped Sr₃MgSi₂O₈ phosphor with good thermal stability. RSC Adv 8(28):15587–15594. 10.1039/C8RA15460F 35539457 10.1039/c8ra00526e PMC9080110

[14] Cullity BD, Stock SR (2001) Elements of X-ray diffraction, 3rd edn. Prentice Hall

[15] Kaczmarek AM, Van Hecke K, Van Deun R, Binnemans K (2012) Effect of dopants on the local environment of silicate-based phosphors. J Lumin 132(12):2760–2765. 10.1016/j.jlumin.2012.06.032

[16] Wang ZL, Chen XY, Zhang JH, Liu Y (2015) Impact of rare-earth ion doping on the local structure of silicate phosphors. Mater Sci Eng B 193:114–120. 10.1016/j.mseb.2015.04.008

[17] Sharma S, Dubey SK, Diwakar AK (2017) Influence of different synthesis methods on structure, morphology, and luminescent properties of BiOCl:Eu³⁺ phosphors. J Lumin 191:88–93. 10.1016/j.jlumin.2017.04.015

[18] Zhu X, Li Y, Li S (2019) Effect of particle sizes and mass ratios of a phosphor on light color uniformity in laminated white leds. RSC Adv 9(4):2112–2117. 10.1039/C8RA09112F 10.1039/c9ra05503g PMC9070674 35529217

[19] Yang L, Dai N, Liu Z, Jiang Z, Peng J, Li H, Li J, Yamashita M, Akai T (2011) Tailoring of clusters of active ions in sintered nanoporous silica glass for white light luminescence. J Mater Chem 21(17):6274. 10.1039/C1JM10374F

[20] Nersisyan SH, Won HI, Won CW, Kirakosyan AG, Jeon DY (2012) Solid combustion wave with two successive reactions to produce phosphor powders. Chem Eng J 198–199:449–456. 10.1016/j.cej.2012.05.033

[21] Lu F-C, Yan-Min GS-QYZ-PY, Pan-Lai Y, Xu L, L., Quan-Lin L (2012) J Alloys Compd 521:77

[22] Kiliç Dokan F, Kuru M, Özlü Torun H, Öztürk E (2023) The effect of different molar ratios of CeO₂ on photoluminescence properties of CeO₂/TiO₂ nanoparticles. Indian J Pure Appl Phys 61:190–197

[23] Sun X, Wen J, Guo Q, Pang F, Chen Z, Luo Y, Peng G, Wang T (n.d.). Fluorescence properties and energy level structure of Ce-doped silica fiber materials. Opt Mater Express, *7*(5), 751–761. 10.1364/OME.7.000751

[24] Arjoca S, VÍllora EG, Inomata D, Aoki K, Sugahara Y, Shimamura K (2015) Temperature dependence of Ce single-crystal phosphors for high-brightness white LEDs/LDs. Mater Res Express 2(5):055503. 10.1088/2053-1591/2/5/055503

[25] Wang Q, Wu W, Zhang J, Zhu G, Cong R (2019) Formation, photoluminescence, and ferromagnetic characterization of Ce-doped AlN hierarchical nanostructures. J Alloys Compd 775:498–502. 10.1016/j.jallcom.2018.09.256

[26] Elmas F, Kırkgeçit R, Özlü Torun H, Öztürk E (2023) Investigation of photochemical properties of CeO₂:0.1Nd and CeO₂:0.05Nd₀.₅M (M: Dy, Sm, Tb). J Photochem Photobiology A: Chem 439:114616. 10.1016/j.jphotochem.2023.114616

[27] Etafo NO, Oliva J, García CR, Mtz-Enríquez AI, Ruiz JI, Avalos-Belmontes F, López-Badillo CM, Gómez-Solis C (2022) Enhancing of the Blue/NIR emission of novel BaLaAlO₄:Yb³⁺(X mol%), Tm³⁺ (0.5 mol%) upconversion phosphors with the Yb³⁺ concentration (X = 0.5 to 6). Inorg Chem Commun 137:109192. 10.1016/j.inoche.2021.109192

[28] Etafo NO (2024) Advancements of lanthanide-doped phosphors in solid-state lighting applications. 1:e220124225904. 10.1002/ajic.202400060

[29] Etafo NO, Oliva J, Garcia CR, Ramirez RCT, Viesca-Villanueva E, Almanza JLF (2024) Blue-Emitting SrLaAlO₄:Ce phosphors obtained by combustion synthesis. Mater Sci Forum 1112:131–137. 10.4028/p-5A41Hx

[30] Lokeswara Reddy GV, Moorthy LR, Jamalaiah BC, Sasikala T (2013) Preparation, structural and luminescent properties of YAl₃(BO₃)₄³⁺ phosphor for white Light-Emission under UV excitation. Ceram Int 39(7):2675–2682. 10.1016/j.ceramint.2012.11.050

[31] Lokeswara Reddy GV, Moorthy LR, Packiyaraj P, Jamalaiah BC (2013) Optical characterization of YAl₃(BO₃)₄ ³⁺–Tm³⁺ phosphors under near UV excitation. Opt Mater 35(12):2138–2145. 10.1016/j.optmat.2013.05.003

[32] Muniz CN, Patel H, Fasta DB, Rohwer LES, Reinheimer EW, Dolgosa M, Graham MW, Nyman M (2018) Rare Earth niobate coordination polymers. J Solid State Chem 259:48–56. 10.1016/j.jssc.2018.02.021

[33] Devi L, Basavapoornima C, Venkatramu V, Babu P, Jayasankar CK (2017) Synthesis of Ca₂SiO₄³⁺ phosphors from agricultural waste for Solid-State lighting applications. Ceram Int 43(16):16622–16627. 10.1016/j.ceramint.2017.07.215

[34] Özlü Torun H, Kırkgeçit R, Kılıç Dokan F, Öztürk E (2021) Preparation of La-Dy-CeO₂ ternary compound: examination of photocatalytic and photoluminescence properties. J Photochem Photobiol A 418:113338. 10.1016/j.jphotochem.2021.113338

[35] Öztürk E, Özpozan NK, Dayan S (2015) Novel Red-Emitting phosphors, (Mg₁₋ₓ₋MnₓDy)Al₂Si₂O₈ and (Mg₁₋ₓ₋MnₓTm)Al₂Si₂O₈. J Therm Anal Calorim 117(2):573–578. 10.1007/s10973-014-4289-7

[36] Bakr M, Kaynar ÜH, Ayvacikli M, Benourdja S, Karabulut Y, Hammoudeh A (2020) Synthesis and competitive luminescence quenching mechanism of Ca₃Al₂O₆:Ln³⁺ (Ln: dy and Sm) phosphors. Mater Res Bull 132:111010

[37] Singh J, Baitha PK, Manam J (2015) Influence of heat treatment on the structural and optical properties of SrGd₂O₄³⁺ phosphor. J Rare Earths 33(10):1040. 10.1016/S1002-0721(15)63750-9

[38] Zhang J, Wang Y, Guo L, Huang Y (2012) Vacuum Ultraviolet–Ultraviolet, X-ray, and Near-Infrared excited luminescence properties of SrR₂O₄³⁺ (R = Y and Gd; RE = Tb, Eu, Yb, Tm, Er, and Ho). J Am Ceram Soc 95(1):243–249. 10.1111/j.1551-2916.2011.04756.x

[39] Öztürk E, Sarılmaz E (2020) The investigation of the photoluminescent and piezoelectric effect of eu³⁺ doped Y₂Ti₂O₇ and Sm₂Ti₂O₇ host crystals. Mater Chem Phys 239:122085. 10.1016/j.matchemphys.2019.122085

[40] Öztürk E, Sarılmaz E (2019) Investigation of photoluminescence and piezoelectric behaviour of the multifunctional Eu₂Ti₂O₇ pyrochlore doped with in³⁺ and Sb³⁺. Mater Res Express 6(10):105710. 10.1088/2053-1591/ab3c0a

[41] Sun X-Y, Han T-T, Wu D-L, Xiao F, Zhou S-L, Yang Q-M, Zhong J-P (2018) Investigation on luminescence properties of Dy³⁺-, Eu³⁺-Doped, and Eu³⁺/Dy³⁺-Co-doped SrGd₂O₄ phosphors. J Lumin 204:89–94. 10.1016/j.jlumin.2018.01.008

[42] Etafo NO (2024) A novel warm Red-Emission of SrLa₀.₉Eu₀.₁AlO₄ phosphor obtained by combustion method. J Eng Res 4(1):1–15. 10.22533/at.ed.1317412409015

[43] McCamy CS (1992) Correlated color temperature as an explicit function of chromaticity coordinates. Color Res Application 17(2):142–144. 10.1002/col.5080170211

